# Active Free Radical Cold Atmosphere Plasma Promotes Healing of Diabetes Foot Ulcer: Mechanism and Challenge

**DOI:** 10.1002/dmrr.70216

**Published:** 2026-08-04

**Authors:** Yiran Wang, Junxiao Zhang, Jinting Yan, Wenhua Bi, Jinlong Ma

**Affiliations:** ^1^ School of Pharmacy Shandong Second Medical University Weifang China; ^2^ Weifang Hospital of Traditional Chinese Medicine, Shandong Second Medical University Weifang China; ^3^ Shandong Engineering Research Center for Smart Materials and Regenerative Medicine Shandong Second Medical University Weifang Shandong China

**Keywords:** angiogenesis, antibacterial, cold atmosphere plasma, diabetic foot ulcers, inflammation, RONS, wound healing

## Abstract

**Background:**

Diabetic foot ulcers (DFUs) have become an important cause of disability and death in patients with diabetes. Traditional therapies are prone to problems such as recurrence and drug resistance, which result in unsatisfactory healing effects for DFUs. Although antibiotics are not the mainstay of treatment for DFUs, antibiotic resistance poses a critical clinical challenge for DFU patients who require long‐term antibiotic therapy.

**Main Text:**

This review summarises the mechanism by which Cold Atmosphere Plasma (CAP) kills bacteria and activates healing ability through the production of reactive oxygen/nitrogen species (RONS). RONS, as the key active species of CAP, exert antibacterial effects in a series of bacterial, cellular, and animal experiments, promote cell migration and proliferation, stimulate angiogenesis, and exert anti‐inflammatory effects. However, current high‐quality clinical trials are still lacking, leaving substantial room for further clinical exploration of CAP in the treatment of DFUs. Finally, the challenges and prospects of CAP in treating diabetic foot ulcers are summarised.

**Conclusions:**

As a comprehensive and innovative treatment method, the RONS produced by CAP offer a potential approach for treating DFUs and serve as a tool for promoting their healing. However, more clinical studies are needed for further verification.

**Abbreviations**
Ang‐1: angiopoietin‐1
APPJ: atmospheric pressure plasma jet
CAP: Cold Atmosphere Plasma
DBD: dielectric barrier discharge
DFUs: diabetic foot ulcers
ECM: extracellular matrix
eNOS: endothelial nitric oxide synthase
EPS: extracellular polymers
FAK: Adhesion kinase
FAs: focal adhesion
FBs: ibrous adhesion
FGF‐2: fibroblast growth factor‐2
FXs: focal complexes
HIS: hyperspectral imaging
MMPs: matrix metalloproteinases
N_2_O_3_
: dinitrogen trioxide
NO: nitrogen monoxide
NO_2_
: nitrogen dioxide
Nrf2: Nuclear factor erythroid 2‐related factor 2
QS: quorum‐sensing
RNS: Reactive Nitrogen Species
RONS: Reactive Oxygen and Nitrogen Species
ROS: Reactive Oxygen Species
StO_2_
: tissue oxygenation
T1D: Type 1 Diabetes
THI: tissue haemoglobin index
TIMPs: mechanistic tissue inhibitors of metalloproteinases
TWI: tissue water index
VEGF: vascular endothelial growth factor

## 1 Introduction

Diabetes mellitus is a serious and incurable chronic disease with a global incidence of more than 500 million [1]. It is expected that the number of people living with type 1 diabetes (T1D) alone will reach 9.5 million by 2025. The number of people with diabetes will grow to 783.2 million by 2045 [2, 3]. The resulting diabetic foot ulcers (DFUs) have become one of the leading causes of amputation [4].

DFUs are different from normal wounds. In normal wounds, the epidermis is continuous, with a sufficient number of fibroblasts that can proliferate and migrate in response to time [5]. The vascular network is dense and provides adequate oxygen and nutrition [6]. In DFUs, an alkaline shift in wound pH leads to the formation of bacterial biofilm, and bacteria continue to colonise the wound. Such bacterial colonisation is disadvantageous, as heavy colonisation can progress to infection, which may cause systemic complications in DFUs [7]. Although this infection is not directly related to the pathophysiology of DFUs, its impact on wound healing is crucial [8]. Sparse blood vessels lead to hypoxia. High levels of pro‐inflammatory cytokines can lead to chronic inflammation, abnormal accumulation and remodelling of extracellular matrix (ECM) [9], which eventually leads to disability and death [10, 11].

On the basis of blood glucose control, conventional therapies for DFUs, such as debridement, offloading, and antibiotics, often have limitations [12, 13], including a high risk of infection recurrence, rapid development of drug resistance, and poor healing effects, resulting in unsatisfactory therapeutic outcomes. Therefore, there is an urgent need to explore healing intervention strategies for DFUs with good antibacterial properties and natural wound healing capabilities by activating their own healing ability.

**FIGURE 1:**
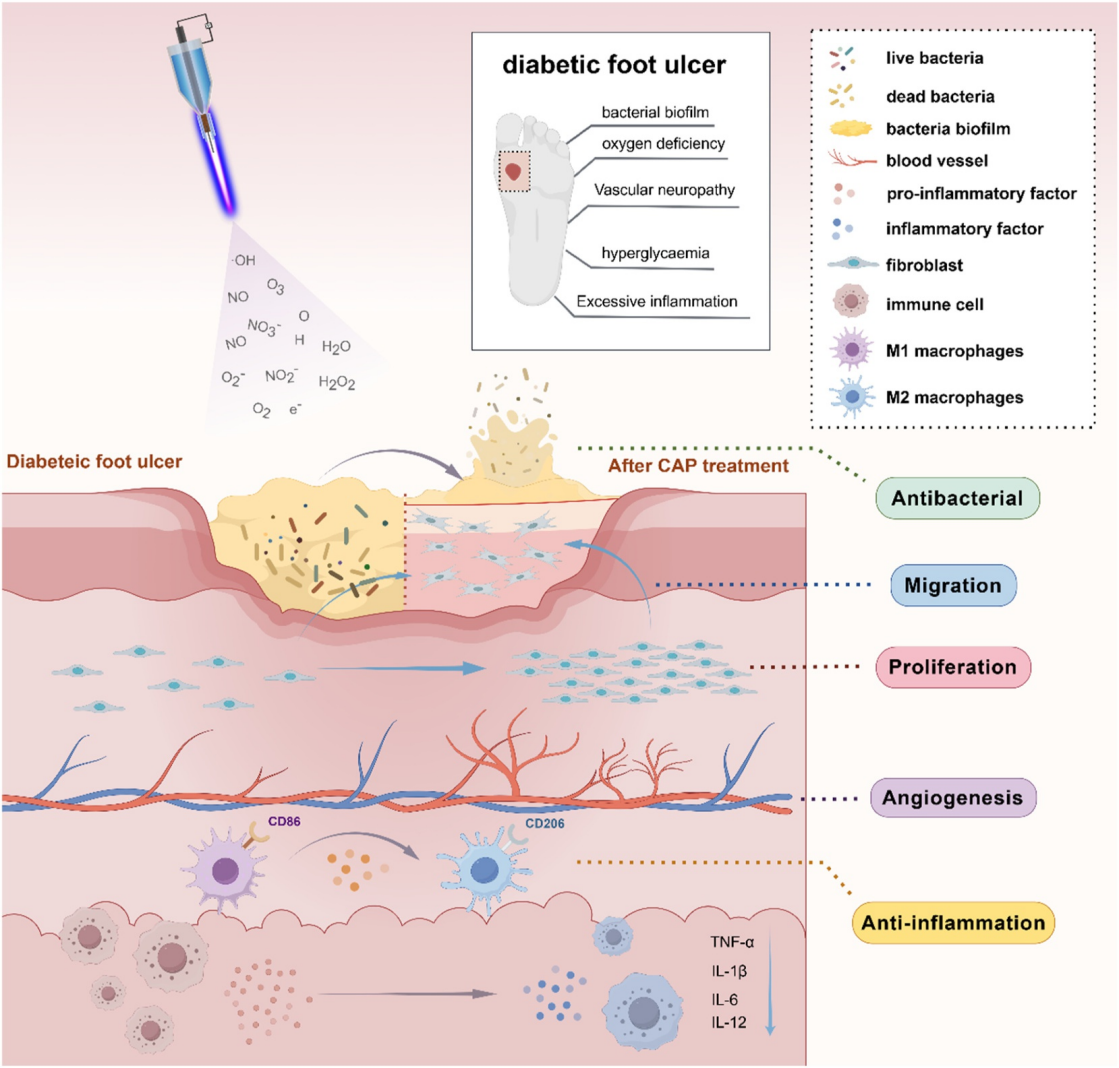
Schematic diagram of the mechanism by which CAP promotes the healing of DFUs. By Figdraw.

Fortunately, in recent years, Cold Atmosphere Plasma (CAP) has emerged as a cutting‐edge interdisciplinary field at the intersection of physics and clinical medicine. Due to its excellent antibacterial properties and ability to promote natural healing, it shows great potential in the field of wound healing treatment. It can kill most bacteria and remove surface biofilms, while ensuring that resistance does not develop [14, 15]. More importantly is that it can produce Reactive Oxygen and Nitrogen Species (RONS) as signal stimulation, activate their own healing ability, promote the process of normal wound healing, and then realize the wound's ‘dynamic and orderly’ process of natural healing (Figure 1). However, CAP treatment for DFUs still has problems such as insufficient clinical trial data, inconsistent treatment equipment and parameters, high‐induced cost and insufficient accessibility. Therefore, this review aims to summarise it more systematically and comprehensively.

## 2 CAP Technique

### 2.1 Active Substances and Functions of CAP

CAP is the fourth state of a gas, containing fully or partially ionised particles [10]. By reacting with the surrounding air, it leads to the partial dissociation and ionization of the surrounding O_2_, N_2,_ and H_2_O, thus producing a series of active substances, such as Reactive Oxygen Species (ROS), such as O_3_, H_2_O_2_, O_2_^−^, etc. Reactive Nitrogen Species (RNS), such as NO, NO_2_, N_2_O_3_, etc. UV, and electric fields [16]. Among them, ROS and RNS play a key role in DFU healing.

CAP is recognized as the fourth state of a gas, comprising fully or partially ionised particles. When CAP interacts with the surrounding air, it leads to the partial dissociation and ionization of O_2_, N_2_, and H_2_O. This interaction results in the creation of various active substances, including ROS such as ozone (O_3_), hydrogen peroxide (H_2_O_2_), and superoxide (O_2_^−^), along with RNS like nitrogen monoxide (NO), nitrogen dioxide (NO_2_), and dinitrogen trioxide (N_2_O_3_). Additionally, the process generates UV light and electric fields. Notably, ROS and RNS are instrumental in the healing process of DFUs.

ROS and RNS play different functions in the treatment of DFUs. The report by Schmidt et al. states that H_2_O_2_ can promote the transformation of macrophages from M1 to M2, reduce the concentration of pro‐inflammatory factors, and finally alleviate the chronic inflammation of DFUs [17]. At the same time, ROS has a significant effect on antibacterial [18, 19, 20]. The peroxynitrite anion (ONOO^−^) is an RNS that is associated with a decrease in pH value [20]. However, the most important role of RNS lies in its active component NO: it can significantly promote angiogenesis and cell migration [21], alleviate the symptoms of ischaemia in the plantar region of DFUs, and act as a signalling molecule to activate a series of signalling pathways. Thus, it can promote DFU wound healing [22].

### 2.2 CAP Equipment

According to the discharge technique and function way, the main use of CAP equipment is divided into three categories respectively is the dielectric barrier discharge (DBD) devices, the atmospheric pressure plasma jet (APPJ) equipment, and microwave plasma torch equipment.

**FIGURE 2:**
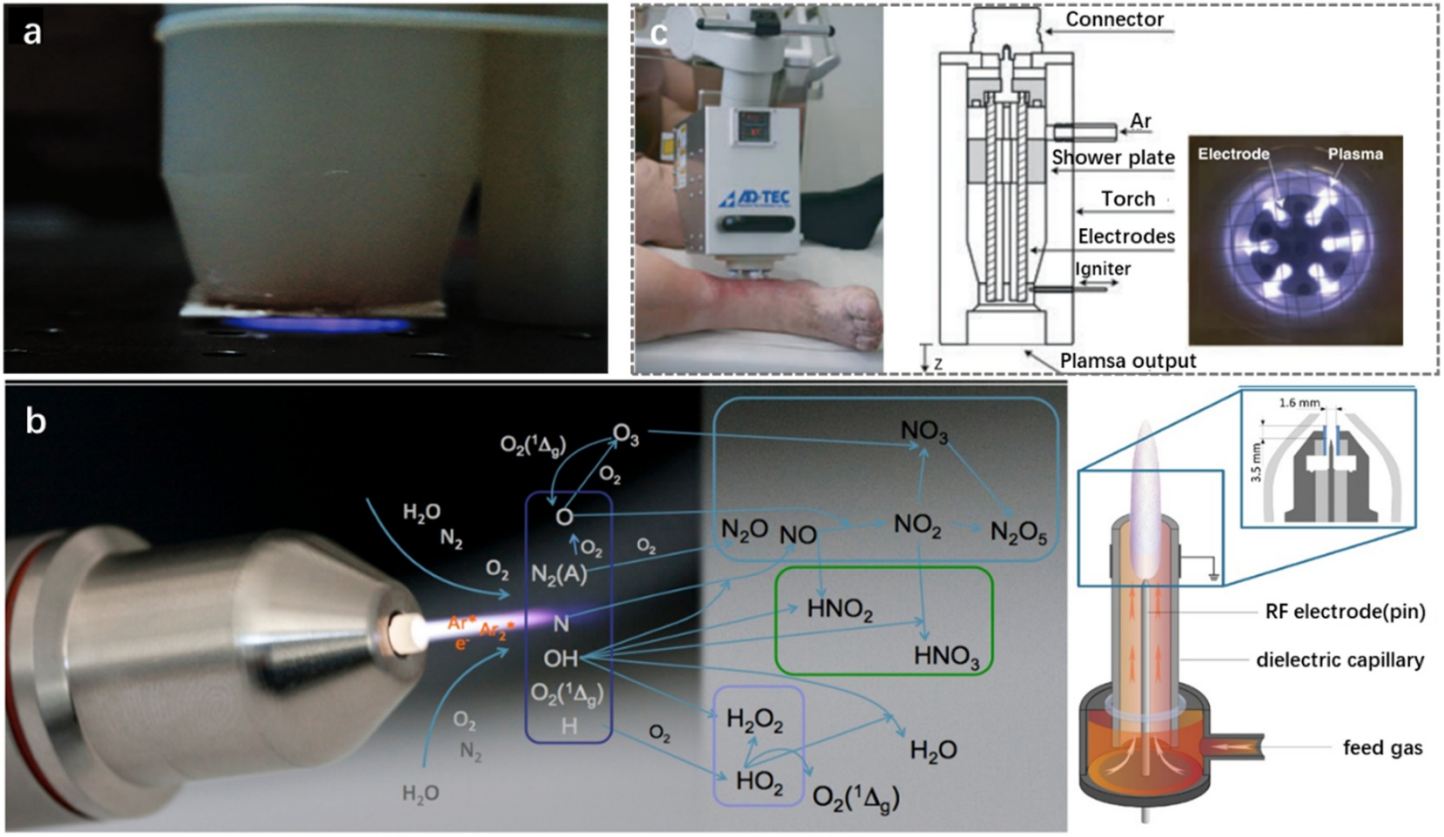
(a) Schematic diagram of the FE‐DBD device, (Reprinted from Ref. [24] with permission. Copyright 2017 Deutsche Dermatologische Gesellschaft (DDG). Published by John Wiley & Sons Ltd.) (b) schematic diagram of APPJ (left) and internal structure (right) (Reprinted from Ref. [25] with permission. Copyright 2018 The Author(s). Published by IOP Publishing Ltd.), (c) device diagram of MicroPlaSter plasma (left) and internal structure (right). (Reprinted from Ref. [26] with permission. Copyright 2010 The Authors. Journal Compilation Copyright 2010 British Association of Dermatologists.).

As the most commonly used device in DBD [23] (Figure 2a), FE‐DBD is characterised by its ability to generate uniform RONS active substances and easily controllable plasma components. However, a constant distance needs to be maintained during the treatment process [23], which makes it more suitable for affected areas of DFUs with fewer skin wrinkles, such as the dorsum of the foot.

A schematic representation of the APPJ represented by kINPen is shown in Figure 2b. Its shape is similar to that of a pen, and it can emit plasma plumes of 9–13 mm. The plumes are about 1.6 mm in diameter and are relatively slender, so they can be used for wounds in more delicate parts of DFUs, such as nail grooves and toe cracks. Moreover, the high concentration of RONS and other charged particles produced by it is also one of its advantages [25].

The MicroPlaSter currently has two models, MicroPlaSter α and MicroPlaSter β, as shown in Figure 2c. As the second‐generation device, MicroPlaSter β has a large four‐joint removable treatment leg, and the plasma torch produced can move at any Angle, which is suitable for the treatment of large plantar ulcer wounds [26]. Due to its strong antibacterial effect, it is currently mainly used for sterilisation and disinfection in clinical trials [27]. Different plasma devices have different therapeutic effects in the treatment of DFUs, as shown in Table 1.

**TABLE 1:** Compares the different roles played by different plasma devices in clinical.

Plasma devices	Gas	Time	The status of the DFUs	Main findings	Ref.
APPJ	He	5 min	Infected	CAP group: 77.3% (CAP score ≤ 0.5), significantly higher than the control group (36.4%)	[28]
MicroPlaSter α	Ar	5 min	Infected	Bacterial load reduced by 34%	[26]
The plasma jet system	He	1 min/cm^2^	Infected	The exudate from the wound has significantly decreased, and the size and grading of the wound have also decreased	[29]
kINPen Med	Ar	30 s/cm^2^	Infected	CAP (53%) versus placebo (50%): Both can reduce microbial load (with no significant difference) and promote wound healing	[30]
Plasma jet	He	180 s	Infected	Complete healing of a chronic ulcer	[31]
Plasma jet	He	5 min	Infected	Effective for wound healing. The levels of inflammatory cytokines decrease, and the average score of bacterial load counts significantly decreases	[32]
CPTcube/CPT patch system (DBD)	Air	2 min	Colonised	Wound closure coefficient > 210%	[33]

It can be seen that CAP can promote the healing of DFUs, but the treatment effect of different devices is different because of the different intake of air. For example, MicroPlaSterα showed excellent antibacterial ability, and kINPen Med showed no significant difference in antibacterial efficacy compared with placebo. DBD can promote wound closure, and the plasma jet with He intake can reduce the expression level of inflammatory factors, thereby promoting wound healing.

## 3 Mechanism of CAP in the Treatment of DFUs

### 3.1 Kill Bacteria

As most of the bacteria infected by DFUs are *Staphylococcus* (such as *methicillin‐resistant Staphylococcus aureus*, MRSA), *Staphylococcus aureus, Escherichia coli*, *Streptococcus*, *Pseudomonas,* and *Pseudomonas aeruginosa* [34], most of the bacteria here can form defensive and protective biofilms in the wound [10, 35]. Furthermore, antibiotics cannot act on biofilms [36], resulting in poor treatment outcomes. More importantly, frequent use of antibiotics can lead to the development of ‘resistance’ in bacteria, causing such infections to recur. Meanwhile, due to the long‐term hyperglycaemic state of DFUs, the pH value of the wound surface is approximately 8.5 [34]. Furthermore, the low‐oxygen wound microenvironment enhances the proliferation and virulence of the main pathogens—anaerobic and facultative anaerobic bacteria—in DFUs, thereby resulting in a postoperative recurrence rate of up to 39.8% in clinical cases [37].

In the 1990s, CAP gradually gained attention due to its efficient bactericidal properties and lack of resistance [36]. Compared with antibiotics and other traditional antibacterial drugs, CAP has unique advantages. It is known that DFU healing faces three major challenges: the biofilm barrier, drug‐resistant bacteria, and a special microenvironment. CAP can exert its effects through active components such as ROS, RNS, and electric fields. Its advantages lie in its ability to penetrate the biofilm, lower the pH value on the skin surface, and not develop drug resistance. These characteristics enable CAP to effectively reduce the number of bacteria on the DFU surface, and it has broad‐spectrum and non‐specific sterilisation properties, capable of killing both harmful pathogenic bacteria and non‐pathogenic bacteria in the wound [38].

Although CAP exhibits broad‐spectrum and non‐specific antibacterial properties, it is relatively more effective against *Staphylococcus aureus* and *E. coli*. In the experiments with bacteria in vitro conducted by Mazandarani et al., the researchers applied CAP treatment for 5 minutes, 7 minutes, and 10 minutes on the plates containing 1.5 × 10^3^ CFU/mL of *Staphylococcus aureus*. The results showed that the bacterial count decreased as the treatment time increased, and the bacterial load dropped to 0 after 10 minutes of treatment. This not only proved that CAP has a good inactivation effect on *Staphylococcus aureus* but also further indicated that its sterilisation effect is time‐dependent [39].

Compared with *Staphylococcus aureus*, *Escherichia coli* and *P. aeruginosa* were more sensitive to CAP. In the bacterial experiments of Jin et al., after 60s of CAP treatment of *P. aeruginosa* and *E. coli,* as well as *Staphylococcus aureus* and *Pseudomonas intermedia*, only *Pseudomonas aeruginosa* and *E. coli,* belonging to gram‐negative bacteria, were completely killed [40]. The reason for this different sensitivity to CAP is that the cell wall of Gram‐positive bacteria is thicker (20–80 nm), which is not easy to be destroyed, while the cell wall of Gram‐negative bacteria is relatively thin (< 10 nm), which is easy to be destroyed, leading to cell lysis [41]. This can indicate that the antibacterial effect is affected by the mechanical properties of the bacteria [15].

It is worth noting that currently, the above sterilisation effects only exist at the level of in vitro experiments, and have not yet been verified in human experiments. In addition to its ability to inactivate common pathogenic bacteria, CAP can also affect other types of bacteria. The specific types of bacteria (include pathogenic bacteria and non‐pathogenic bacteria) and the sterilisation effect are shown in Table 2 below.

**TABLE 2:** CAP can inactivate various bacteria.

Bacteria	Plasma source	Gas	Handling time	Antibacterial effect	Ref.
*Staphylococcus aureus*	APPJ	Air	10 min	1.5 × 10^3^ CFU/mL decreased to 0	[39]
*MRSA*	Plasma‐activated saline	Air	24 h	Reduce > 2 log CFU/mL	[42]
*Enterococcus faecalis*	APPJ	Ar	6 min	Reduce 1.9 log CFU/mL	[43]
*Streptococcus*	MicroPlaSter β	Ar	5 and 10 min	17% and 5% of bacteria survive	[44]
*Mycobacterium tuberculosis*	NTPJ (non‐thermal plasma jet)	N_2_ + H_2_O	5 min	98% of bacteria are killed	[45]
*Klebsiella pneumoniae*	APPJ	Ar	5 s	Completely eradicate	[45]
*Pseudomonas aeruginosa*	DC‐driven CAP	Air	2 min	Reduce 5 log CFU/mL	[46]
*Acinetobacter baumannii*	APPJ	Air	1.5 min	Reduce 5–5.5 log CFU/mL	[47]
*Propionibacterium acnes*	NAPJ and NSPJ	Ar/air	1800 s/600 s	CFU is 0	[48]
*Listeria monocytogenes*	DBD	He + O_2_	2.5 min	Reduce 3.6 log (CFU/cm^2^)	[49]
*Salmonella enteritidis*	Gliding arc discharge plasma	Air	10 min	< 2.0 CFU/mL	[50]
*Klebsiella pneumoniae*	APPJ	Ar	5 s	Completely eradicate it	[51]
*Pseudomonas fluorescens*	APPJ	Ar	15 min	Reduce 1.77 log_10_ CFU mL^−1^	[38]
*Bacillus subtilis*	APPJ	Ar	15 min	Reduce 1.58 log_10_ CFU mL^−1^	[38]
*Lactiplantibacillus plantarum*	APPJ	Ar	15 min	Reduce 1.31 log_10_ CFU mL^−1^	[38]

### 3.2 The Mechanism of Killing Bacteria

The formation of biofilms is one of the key factors contributing to the high recurrence rate and drug resistance of DFUs. Its dense structure is known to be maintained by extracellular polymers (EPS) [52]. CAP leads to the destruction of EPS through the peroxidation chain reaction of EPS lipids, modification and degradation of proteins, and chemical bond breaking of carbohydrates [53]. CAP can also inhibit the formation and regeneration of biofilm by inhibiting the quorum‐sensing (QS) system [54]. The experimental results of Cui et al. showed that CAP treatment resulted in differential down‐regulation of the expression of QS‐related genes in bacteria, which suggested that CAP could interfere with the synthesis of QS signalling molecules and affect the formation of bacterial biofilm [55]. After biofilm rupture, the resistant bacteria are exposed, and the CAP can act more efficiently.

Unlike traditional antibiotics, which only act on a single target and easily cause drug resistance, CAP acts on the cell wall, cell membrane, protein, and DNA at the same time to avoid the evolution of drug resistance genes of drug‐resistant bacteria. One of the mechanisms for killing bacteria is the leakage of cell contents due to the destruction or permeabilisation of the cell wall and cell membrane. ROS produced by CAP is considered a traditional bactericidal factor [15], in which O_3_ can oxidise the bacterial cell wall and permeabilise the cell wall, leading to the death of bacteria [56]. H_2_O_2_ and ·OH, as strong oxidants, can oxidise the cell membrane of bacteria and cause rupture, thus achieving the effect of sterilisation [56].

**FIGURE 3:**
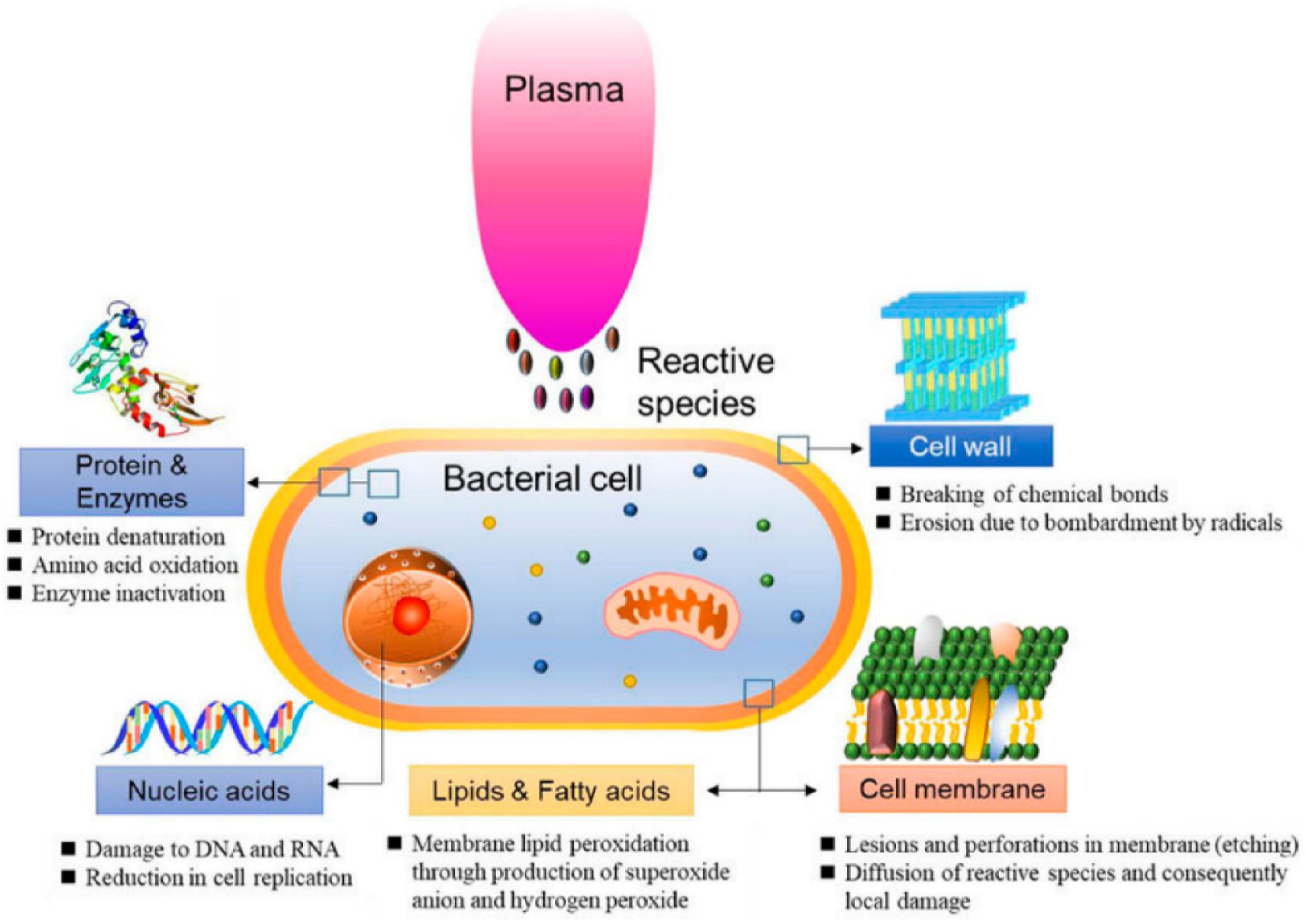
Schematic representation of the CAP sterilisation mechanism. Reprinted from Ref. [62] with permission. Copyright 2023 The Author(s). Published by MDPI.

RONS can also achieve oxidative modification of amino acids, such as hydroxylation, carbonylation, and nitration of amino acids, which terminate the normal function of proteins and cause bacterial death [57]. RONS of CAP can also act on bacterial DNA to inhibit its proliferation. It can cause damage to nucleic acid molecules by causing DNA strand breaks [58]and the reaction products of CAP with other molecules [59]. The electric field generated by CAP cannot kill bacteria directly, but when it acts on the bacterial cell membrane, it forms ‘electrical pores’ on the membrane [60]. This effect makes the active components, such as RONS, easier to enter the bacterial interior, thereby increasing the leakage rate of DNA [61]. Figure 3 shows the action site and mechanism of CAP sterilisation. By acting on the cell wall, cell membrane, protein, and DNA, it is proved that CAP can effectively kill bacteria [62].

In view of the special alkaline environment on the surface of DFUs, NO_2_^−^ in RNS can adjust the pH to 6.5–7.0 [63]. This acidic environment makes it easier for reactive molecules to penetrate the bacterial cell wall and also increases the permeability of the cell membrane. Some studies have also proved that peroxynitrite requires the catalytic decomposition of hydrogen ions to play an antibacterial effect [64], which reverses the proliferation of bacteria in an alkaline environment.

### 3.3 Reduce Inflammation

Excessive and long‐term inflammation is an important cause of poor healing of DFUs [65]. Normal wound healing goes through stages of haemostasis, inflammation, proliferation, and remodelling [66]. However, the high sugar microenvironment of DFUs promotes excessive release of pro‐inflammatory factors. These pro‐inflammatory factors fail to dissipate in time during the healing process, thereby leading to a higher proportion of M1 macrophages in immune cells and other issues [67]. This series of situations disrupts the normal stages of wound healing, ultimately causing the wound to remain in the inflammatory phase for a long time [68].

In view of the over‐expression of pro‐inflammatory factors, the lack of anti‐inflammatory factors, and immune cell imbalance, RONS produced by CAP is like a precise regulator. On the one hand, it regulates anti‐inflammatory factors by simulating natural healing to repair immune cell imbalance, and on the other hand, it affects the cross‐regulation of nuclear factor erythroid 2‐related factor 2 (Nrf2) and NF‐κB pathways at the molecular level to achieve wound healing.

#### 3.3.1 Regulation of Inflammatory Factors

CAP has a regulatory response to inflammatory factors at different stages of inflammation. Zhao et al. conducted the experiment by creating a mouse model of DFUs. It has been proved that CAP treatment in the early stage of inflammation (0–3 days) could significantly down‐regulate IL‐12, IL‐1b, IL‐17A, and other pro‐inflammatory factors and improve the excessive accumulation of pro‐inflammatory factors. Furthermore, during the middle stage of inflammation (3–7 days), the expression levels of anti‐inflammatory factors such as IL‐1Ra, IL‐4, IL‐5, IL‐10, IL‐13, and TGF‐β significantly increased [63]. This change reversed the adverse environment caused by high concentrations of pro‐inflammatory factors, thereby facilitating the transition of wound healing from the inflammatory stage to the repair stage.

CAP not only directly improves the problem of ‘too much pro‐inflammation and not enough anti‐inflammation’ by regulating inflammatory factors but also provides a signal for the function of immune cells. For example, IL‐4, IL‐5, and IL‐13, as important anti‐inflammatory factors to promote differentiation and maintain M2 cells, are significantly increased after CAP treatment to maintain the balance of M1/M2 macrophage polarization [63].

#### 3.3.2 Promote Macrophage Phenotype Transformation

One of the reasons for excessive inflammation caused by DFUs is the imbalance of M1/M2 macrophage polarization. In the process of normal wound healing, the phenotype of macrophages changes from M1 to M2, which tends to suppress inflammation [69]. On the contrary, this transformation is impaired in DFUs, resulting in a higher proportion of M1 than M2, presenting a tendency to induce inflammation, leading to excessive inflammation, reduced angiogenesis, and other chronic wound problems [69].

Many current studies have shown that CAP treatment can affect the polarization of M1/M2 macrophages. In the study by Crestle et al., the differentiation of human peripheral blood mononuclear cells was investigated using M0 macrophages as the model [70]. M0 macrophages were treated with CAP and cultured in medium supplemented with 20% FBS. The expression of M1/M2 phenotypic markers (CD16/CD86 for M1; CD163/CD206 for M2) was then analysed. The results show that the M1 macrophages (labelled as CD14, CD16, and CD86) in the culture medium decrease with the extension of the treatment time. Meanwhile, the M2 macrophages (labelled as CD14, CD163, and CD206) gradually increased. After 30 seconds of treatment, the decreasing trend of M1 and the increasing trend of M2 become very obvious [70], and this has not yet been verified in human experiments. Zhao et al. conducted an immunohistochemical test and found that on the 2nd and 3rd days after CAP treatment, the expression level of CD206 in the treatment group was significantly higher than that in the control group. From this, it can be concluded that CAP has the effect of promoting the transformation of macrophages to the M2 type [63].

#### 3.3.3 Affect the Cross‐Regulation of Nrf2 and NF‐κB

**FIGURE 4:**
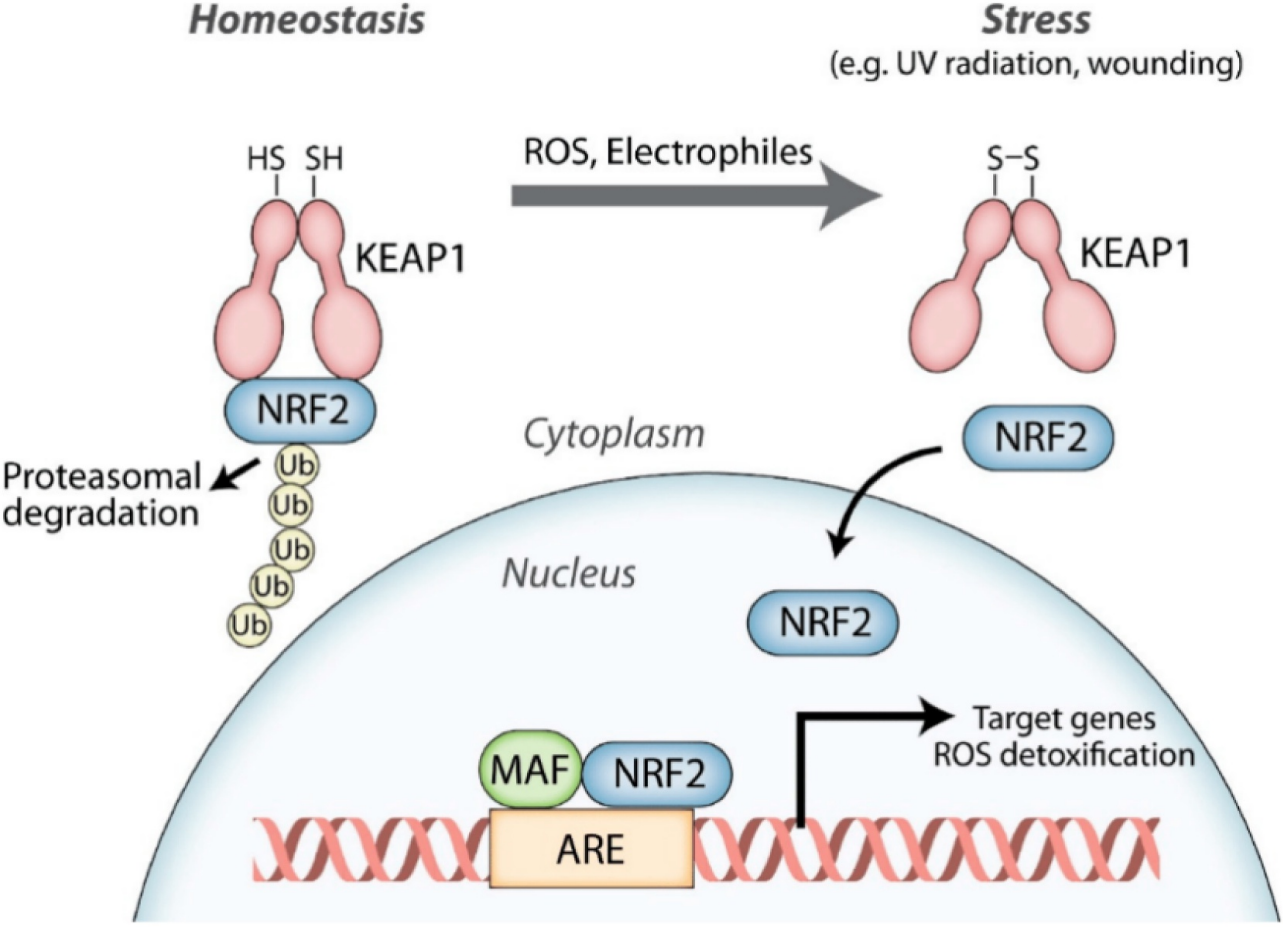
Under steady‐state conditions, Keap1 mediates the degradation of NRF2. However, stress causes a conformational change in Keap1, allowing NF2 to enter the nucleus and bind to ARE to activate ROS detoxification genes, thereby achieving cellular antioxidant regulation. Reprinted from Ref. [71] with permission. Copyright 2019 The Authors. Published by MDPI.

Nrf2 is a transcription factor that plays a crucial role in reducing ROS levels and maintaining the intracellular redox balance during wound healing. It can significantly inhibit chronic inflammation and promote granulation and re‐epithelialisation [71, 72]. The specific Nrf2 signalling pathway is shown in Figure 4. There is a problem of dysregulated Nrf2 signalling pathway in DFUs, which also leads to the spread of inflammation in the wound and the blocked healing [17]. CAP can solve the problem of inflammatory disorders and oxidative stress in DFUs by regulating the Nrf2 signalling pathway and NF‐κB pathway [71].

Nrf2 activation has a significant effect in regulating inflammatory responses. It does not directly inhibit the inflammatory response, but indirectly inhibits the pro‐inflammatory transcription factor NF‐κB by reducing the concentration of ROS [73]. Because ROS binds to the ARE sequences of target genes such as HO‐1 and NQO1, HO‐1 can directly block the nuclear translocation of NF‐κB p65 subunit, and NQO1 inhibits the phosphorylation of IKKβ by reducing intracellular ROS levels, which is known to be the key kinase for NF‐κB pathway activation [74]. Finally, it can reduce the transcription of IL‐1β, TNF‐α, IL‐6, and other pro‐inflammatory factors and inhibit excessive inflammation in DFUs [75, 76]. At the same time, Nrf2 also has an effect on immune cells. For example, in macrophages, Nrf2 can promote their transformation into more anti‐inflammatory M2 cells [77].

**FIGURE 5:**
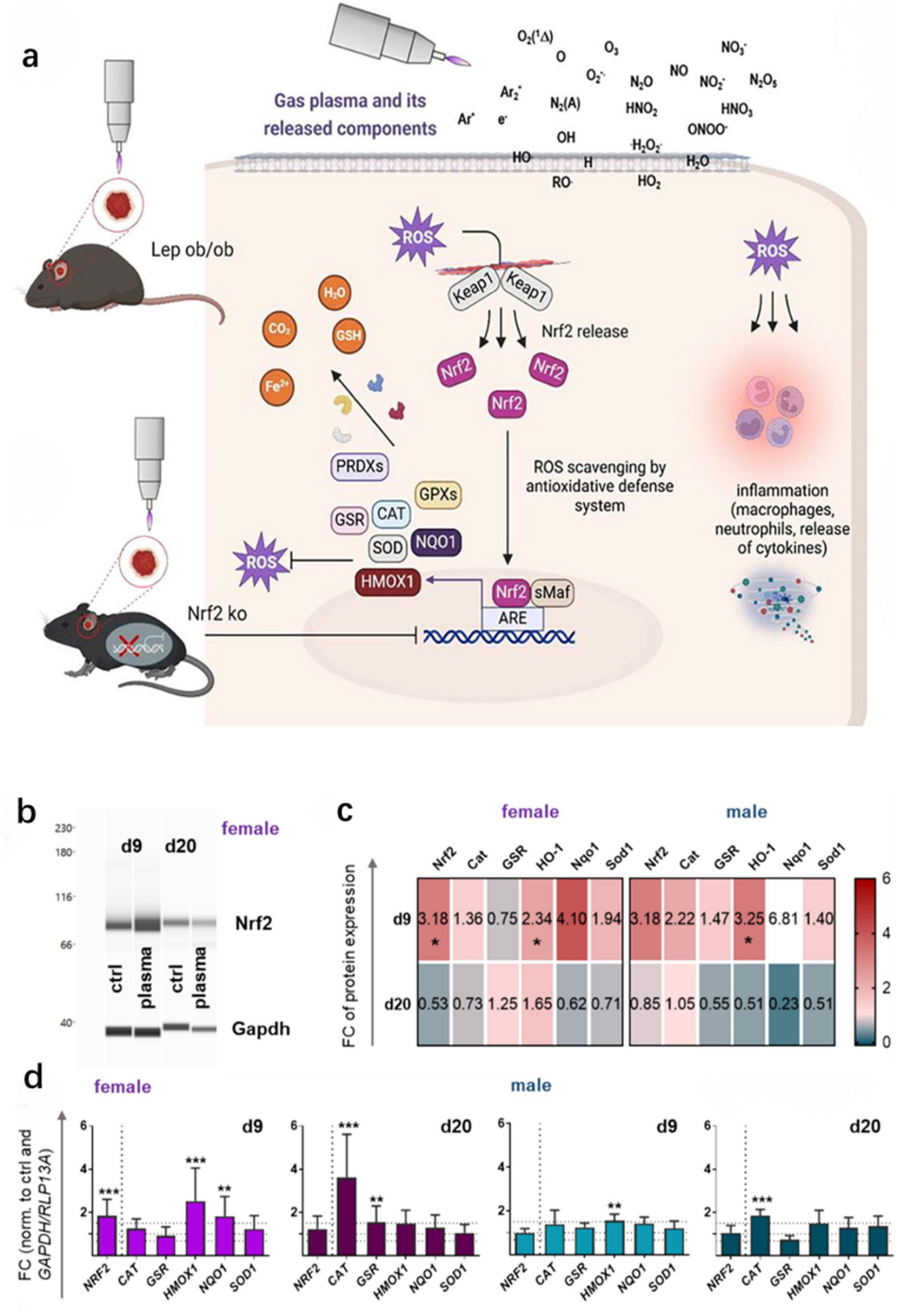
(a) CAP and its components regulate the Nrf2 pathway, thereby influencing oxidative stress (ROS) and inflammatory responses in different mouse models (Lep ob/ob, Nrf2 ko); (b) Images of Nrf2 protein expression in wounds analysed by WES; (c) Detection of Nrf2 and downstream protein expressions at different times and in different genders, as well as quantitative results of related mRNAs. (d) Quantitative mRNA expression of the transcription factor NRF2 and its downstream targets (CAT, GSR, HMOX1, NQO1, SOD1). Reprinted from Ref. [17] with permission. Copyright 2025 The Authors. Published by Elsevier B.V. on behalf of Cairo University.

In the report by Anke Schmidt et al., it was demonstrated that CAP was able to activate Nrf2 and nuclear translocation in diabetic wound sites, and the expression levels of multiple ARE genes regulated downstream of CAP were significantly enhanced and showed the characteristics of synergistic expression (Figure 5a–d). CAP and its components affect the Nrf pathway, as shown in the Figure 5a. However, in the presence of Nrf2, this protective effect against oxidative stress was short‐lived. Representative protein expression analysis using the WES system (Figure 5b,c) showed that Nrf2 expression level increased at day 9 after CAP treatment, but decreased at day 20. This change is consistent with the normal wound healing process: inflammation, and its expression is decreased in the proliferation and remodelling phase to prevent excessive antioxidant stress response, leading to tolerance of cytoprotective mechanisms [17].

### 3.4 CAP Promotes Re‐Epithelialisation of the Wound

One of the reasons for poor wound healing in DFUs is the disorder of re‐epithelialisation, which involves the migration, proliferation and differentiation of epidermal keratinocytes and fibroblasts to cover the exposed wound surface. CAP accelerates wound healing by promoting cell migration and proliferation.

#### 3.4.1 Accelerate the Migration of Keratinocytes

The specific mechanism by which CAP promotes cell migration involves changes in cell adhesion structure and reorganization of the cytoskeleton. Cells form three types of dynamic adhesion complexes with the ECM via integrins: focal complexes (FXs), focal adhesion (FAs), and fibrous adhesion (FBs) [78]. FXs and FAs are dynamic and transient and contribute to cell migration, whereas FBs are stable and persistent, promoting cell anchoring and fibril formation and inhibiting migration. Studies have shown that CAP treatment can inhibit FB formation while promoting FXs/FAs turnover, thereby enhancing cell migration [79]. In the experiments, it was observed that Rac, fibronectin (FN) receptor integrin α5β1, and tensin, which are FB markers, were significantly down‐regulated after CAP treatment, indicating that CAP‐capable treatment could promote cell migration.

Efficient cell migration depends not only on the adhesion structure but also on rapid adhesion dissociation and reorganization. Adhesion kinase (FAK) and Paxillin are the core signalling proteins in the adhesion complex, and their tyrosine phosphorylation is a key signal for adhesion assembly and migration [80]. Adhesion strength is regulated by the aptamer protein Vinculin on the cytoskeleton. Vinculin inactivation or cutting will reduce the strength of adhesion, promote back out and move forward, which is the key factor for cell migration [81].

**FIGURE 6:**
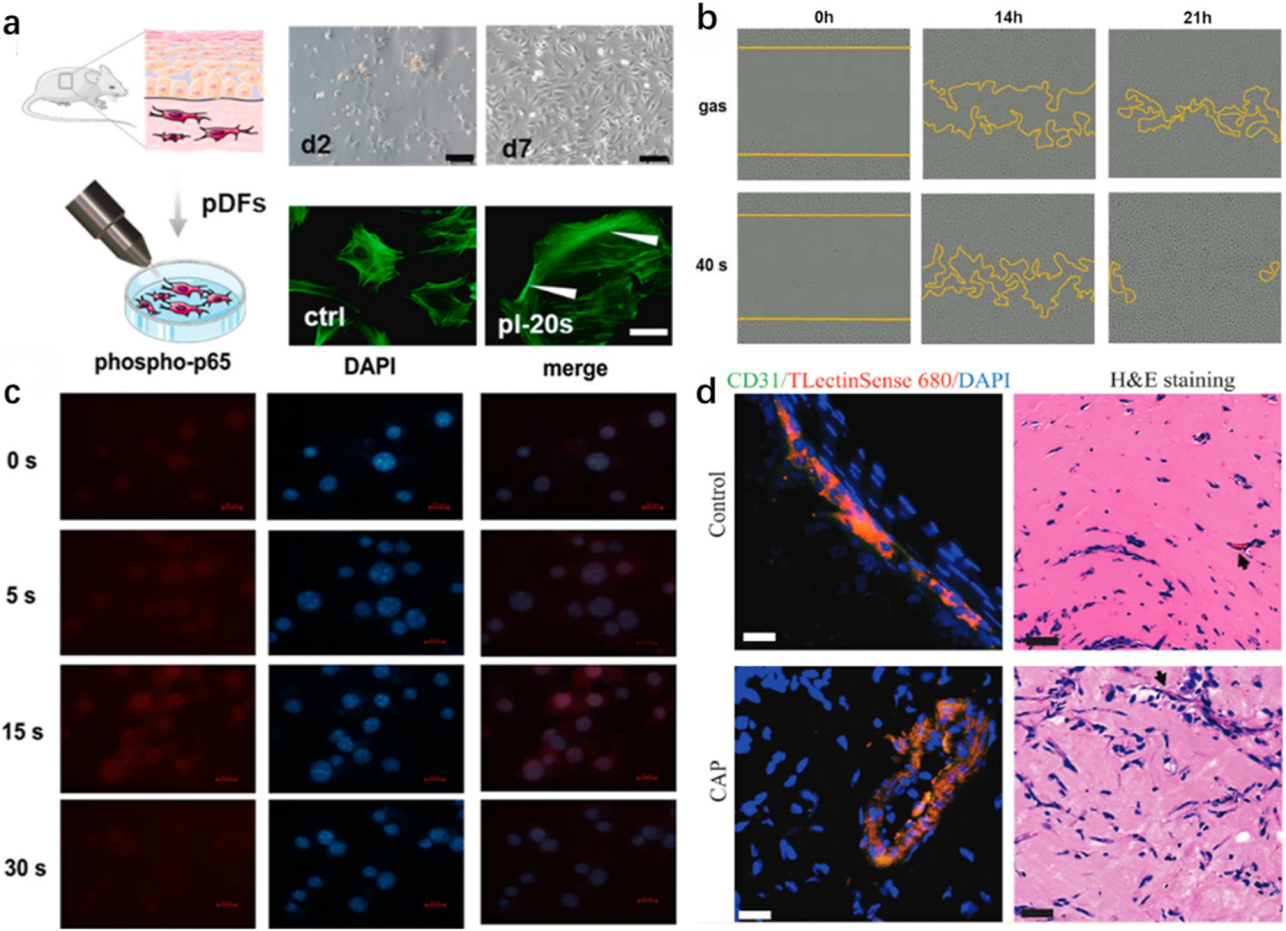
(a) Primary fibroblasts were incubated with CAP‐treated medium for 20, 60, and 180 s. This was followed by F‐actin staining. (Reprinted from Ref. [79] with permission. Copyright 2020 The Author(s). Published by Elsevier B.V.) (b) Yellow lines representing cell edges. After the cells were treated with CAP for 10–60 s, it was observed that the area between the yellow lines gradually became smaller with the extension of time. (Reprinted from Ref. [82] with permission. Copyright 2020 The Author(s). Published by MDPI.) (c) Fluorescence images indicate that the nucleus is blue and phospho‐p65 is red, indicating entry into the nucleus, confirming that CAP activates NF‐κB. (Reprinted from Ref. [83] with permission. Copyright 2017, The Author(s)) (d) Dual immunofluorescence images showing CD31^+^(green) and TLectinSenseTM 680^+^(red), as well as H&E staining results. (Reprinted from Ref. [21] with permission. Copyright 2019 The Author(s) Published by John Wiley and Sons Ltd.).

In addition to adhesion complexes, changes in the cytoskeleton also affect migration. F‐actin staining (Figure 6a) showed that CAP‐treated cells had an altered morphology with more spreading and fewer stress fibres, but enhanced staining at the cell edges (such as pseudopodia and filopodia), indicating that the cells had changed to a ‘migratory phenotype’ in preparation for migration [79].

Many experiments have also demonstrated that CAP is able to promote cell migration. Marches et al. showed that a short time (20–40 s) of helium plasma treatment significantly accelerated keratinocyte migration [82]. Using scratch assays, they observed that keratinocyte migration was significantly enhanced after CAP treatment, whereas longer treatment did not affect scratch wound closure (Figure 6b). Notably, the study also noted that this effect did not cause detectable intracellular oxidative stress, mitochondrial dysfunction, or heat shock response [82]. This finding challenges the traditional view that cellular effects are necessarily mediated by oxidative stress and provides a new direction for further exploring the non‐oxidative stress‐mediated mechanism of CAP‐regulated cellular behaviour.

#### 3.4.2 Affects the Proliferation of Fibroblasts

Fibroblasts play an important role in the proliferation and remodelling phases of wound healing. Promoting the proliferation of fibroblasts can promote the contraction of wounds [84], promote the secretion of cytokines, affect other cells through paracrine pathways [85], and promote the ECM [84]. CAP can promote cell proliferation by activating the NF‐κB pathway and increasing the expression of cyclin D1 through ROS/RNS.

NF‐κB is a nuclear transcription factor that affects the expression of genes involved in inflammation, immune response, and cell proliferation during wound healing [86]. In the process of cell proliferation, the cell cycle transitions from G1 phase to S phase, in which CyclinD1 plays an important role and is an important protein to achieve this phase transition [87]. CAP induces the activation of NF‐κB through the phosphorylation or dephosphorylation and oxidation of thiol residues caused by ROS [88]. Liu et al. demonstrated experimentally that CAP activates the NF‐κB pathway and upregulates cyclin D1 to promote cell proliferation (Figure 6c). It is known that the translocation of phosphorylated p65 from the cytoplasm to the nucleus is considered to be an important sign of activation of the NF‐κB pathway [83].

At present, there are significant differences in the results of different studies on the promotion of cell proliferation by CAP [89, 90]. In the study ‘Supporting the promotion of proliferation’, this effect was attributed to the mild oxidative stress caused by ROS, which induced fibroblast growth factor‐2 (FGF‐2) release from fibroblasts to promote cell proliferation [91]. However, Balzer et al. observed a significant inhibition of fibroblast proliferation in their study using a DBD plasma source [92].

The reason for this result can be explained by the report of Shi et al., who found that CAP promoted cell proliferation with a ‘dose‐dependent’ effect, that is, high concentrations inhibited cell proliferation and low concentrations promoted cell proliferation [93]. This view emphasises the importance of dose as well as equipment, and suggests that the differences between studies may be due to differences in plasma equipment, operating parameters, and cell models. However, it is still unknown at present how this result compares with the results of the in human experiments, as it is currently only at the cellular experimentation stage.

### 3.5 Promote Angiogenesis

Hypoxia is a major feature of many chronic wounds, especially DFUs [94]. Blood vessels are frequently damaged during angiogenesis, the formed granulation tissue is affected, and insufficient blood vessels to supply oxygen impede wound healing [95, 96]. Angiogenesis is a complex process regulated by a variety of growth factors and vascular response mediators. As RNS produced by CAP, NO can directly stimulate endothelial cells to produce vascular endothelial growth factor (VEGF) and other substances to participate in the healing process of DFUs [97], or as an exogenous signalling molecule, promote the production of endogenous NO through the eNOS/NO signalling pathway, thereby affecting blood vessels [82]. In addition, it can also indirectly stimulate endothelial cells to secrete a variety of endogenous growth factors, anti‐growth factors, and vascular response mediators, such as FGF‐2, through paracrine mechanisms, which is the communication between keratinocytes and endothelial cells, as well as between fibroblasts and endothelial cells [97].

VEGF is a key angiogenic factor in the formation of granulation tissue in the proliferative phase [98], which mainly promotes the formation of early blood vessels [99]. At the same time, angiopoietin‐1 (Ang‐1), another important angiogenic factor, can synergistically stimulate the effect of VEGF [100]. The experiments of Badr et al. showed that the concentration of VEGF in diabetic mice treated with CAP was restored to the level of non‐diabetic mice, and the expression of Ang‐1 was significantly increased [22]. The results showed that CAP promoted angiogenesis by increasing the concentrations of VEGF and Ang‐1.

In addition, Duchesne et al. found in their study that CAP treatment significantly increased the phosphorylation of endothelial nitric oxide synthase (eNOS), leading to an increase in endogenous NO. Based on Badr et al., they further explored how VEGF promotes angiogenesis and found that exogenous NO produced by CAP can indirectly promote eNOS phosphorylation and NO production by enhancing the VEGF/VEGFR2 signalling pathway, forming a positive feedback [21]. NO can also enhance the migration and proliferation of endothelial cells and accelerate angiogenesis [101]. All of the above confirmed that NO does play an important role in angiogenesis.

In fact, ROS has a similar effect. Similar to VEGF, FGF‐2 is also an angiogenic factor that activates angiogenesis by directly or indirectly upregulating VEGF in endothelial cells [21]. Related studies have shown that ROS can induce the production of FGF‐2, which causes the proliferation of endothelial cells and the formation of vascular lumen structure, and its main components include hydroxyl free radicals and hydrogen peroxide [102, 103].

CAP has a positive effect on angiogenesis. In the experiments of Duchesne et al., the ability of CAP to significantly promote neovascularisation was confirmed by dual fluorescence images stained with CD31^+^ and TLectinSenseTM 680^+^ as well as H&E sections. As can be seen in Figure 6d, the vessel fluorescence signal was stronger in the CAP‐treated group compared to the control group. H&E sections were used to assess vessel density. Greater vessel density can be found in the CAP‐treated group [21].

**FIGURE 7:**
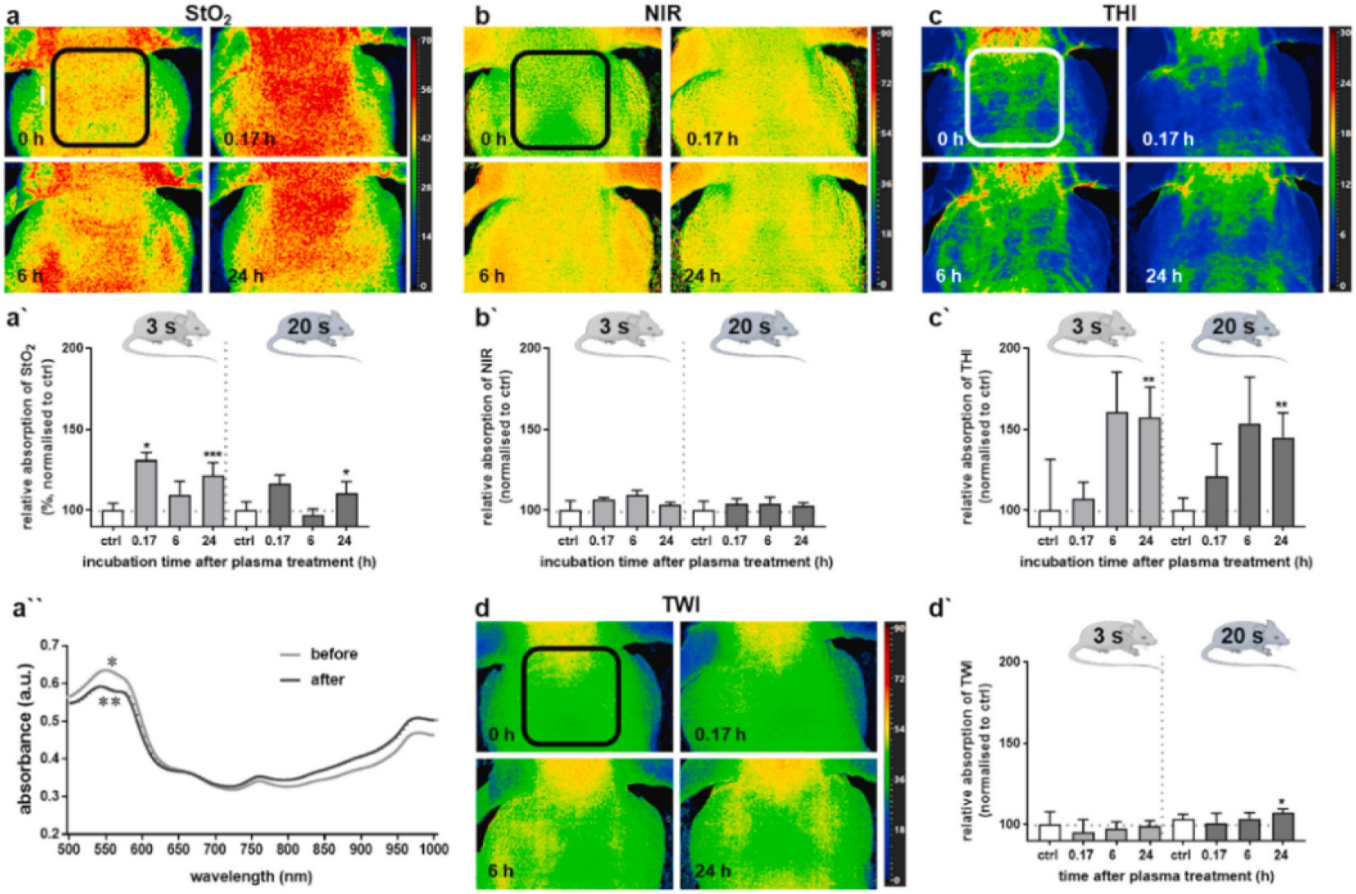
(a–d) Shows colour images of tissue oxygenation (StO2, a) and deep perfusion (NIR, b) as well as tissue haemoglobin index (THI, c) and tissue water index (TWI, d) of mice treated with CAP for 3s. (a'–d') The HIS parameters are quantified. From 550 to 570 nm, it changes from a single peak (*) to a double peak (**), indicating an increase in StO_2_. Reprinted from Ref. [104] with permission. Copyright 2020 The Author(s). Published by Elsevier Inc.

Proper promotion of microcirculation in local areas has become one of the methods to solve the problem of hypoxia. This process is affected by tissue oxygenation and haemoglobin levels. CAP can increase perfusion by increasing tissue oxygenation and haemoglobin levels, improve hypoxic symptoms of wounds, promote angiogenesis, and promote wound healing [104, 105]. Schmidt et al. used a novel hyperspectral imaging (HSI) system to record the tissue haemoglobin index (THI), tissue water index (TWI), and tissue oxygenation (StO_2_) of mice in real time [104]. The results showed that the microcirculation coefficient was different in mice of different genders. In female mice, StO_2_ was significantly enhanced after CAP treatment, while THI and TWI increased less in males than in females after CAP treatment, but both showed an overall promoting effect on microcirculation parameters (Figure 7) [104]. Notably, although gender affected the parameters of microcirculation, the difference was mainly determined by the stage of wound healing.

### 3.6 Promote Tissue Remodelling and Inhibit Scar Formation

#### 3.6.1 Promote Tissue Remodelling

Slow healing of chronic wounds is also associated with ECM imbalance caused by matrix metalloproteinases (MMPs) dysregulation [106]. MMPs are a class of zinc‐dependent proteolytic enzymes capable of degrading damaged ECM [107], and their activity is inhibited by mechanistic tissue inhibitors of metalloproteinases (TIMPs). In normal skin or acute trauma, the expression of MMPs is usually maintained at a low level [108] and plays a role in multiple links of wound healing: degradation of damaged ECM, promotion of recruitment of related cells, re‐promotion of granulation tissue formation, promotion of angiogenesis, and expression of related growth factors [109].

However, in DFUs, MMPs are usually overexpressed [106], leading to excessive ECM degradation and imbalance, thereby delaying wound healing. CAP reduces the expression of MMPs and TIMPs by producing ROS as signalling molecules, restores the balance of ECM degradation, and promotes ECM remodelling and wound healing [110]. Studies have found that after plasma treatment, the expression of MMP2, MT1‐MMP, and TIMP2 is plasma‐dependent and time‐dependent with different wound healing stages: in the inflammatory stage, inhibition of MMP2 activity and up‐regulation of TIMP after CAP treatment are helpful to reduce ECM destruction and maintain the integrity of wound structure. During the proliferative phase, MT1‐MMP and MMP2 are gradually activated to remove the damaged ECM and provide space for the growth of new tissue. During the remodelling phase, TIMP2 is down‐regulated and MMP2 remains active, contributing to the orderly arrangement of collagen fibres and preventing scar formation [79].

#### 3.6.2 Inhibition of Scar Formation

Keloid formation is mainly caused by the abnormal arrangement of type I collagen and type III collagen [111]. The ratio of type I collagen to type III collagen determines whether abnormal cross‐linking reactions and disordered arrangement of fibre bundles will occur in trauma, leading to the formation of fibrous scars. When the ratio is low or collagen is over‐synthesised, the risk of keloid formation increases [100]. α‐SMA is a differentiation marker of myofibroblasts, and its expression level of α‐SMA is usually significantly upregulated in scarred wounds. From the perspective of signalling pathways, the TGF‐β1/Smad2/3 pathway is currently recognized as an important signalling pathway for scar formation [112], which can not only cause excessive synthesis of ECM components but also promote excessive proliferation of fibroblasts, resulting in the production of keloids.

In view of the causes of scar formation mentioned above, previous studies have explored ways to solve these problems. Wang et al. found that CAP could not only accelerate wound healing but also reduce keloid formation by regulating TGF‐β1/Smad2/3 signalling pathway [113]. The results showed that compared with the control group, the expression of TGF‐β1 was significantly down‐regulated in the CAP‐treated group, and the expression levels of phosphorylated Smad2 and Smad3 (*p*‐Smad2 and *p*‐Smad3), which are biologically active forms of Smad2 and Smad3 proteins, were also significantly decreased in the CAP‐treated group. At the same time, down‐regulation of α‐SMA expression is also an effective method to inhibit scar formation [114]. The results showed that the content of α‐SMA in CAP‐treated wound tissue was lower than that in the control group. Oceane Blaise et al. reported that CAP was able to cause collagen I synthesis in wounds. They found that CAP treatment was able to activate dermal fibroblasts to synthesise collagen I and was able to enhance the expression of collagen IV and laminin [115]. These data suggest that CAP can inhibit scar formation by affecting the TGF‐β1/Smad2/3 pathway, reducing the expression level of α‐SMA, and increasing the ratio of collagen type I to collagen type III.

Inhibition of cell proliferation may become an effective strategy for the treatment of hyperproliferative skin diseases, such as excessive scarring [92]. In the study by Balzer et al., it was found that DBD treatment of human skin fibroblasts can reduce the activity and proliferation ability of fibroblasts, and it is speculated that the mechanism of action is mainly caused by HO_2_ produced by DBD. However, considering the whole wound healing process, the practice of solely using DBD to inhibit excessive scarring has certain limitations. Due to the long treatment time of DBD (300 seconds), although the generated HO can inhibit cell proliferation, it will also cause excessive oxidative stress in cells and delay the healing process [92]. Therefore, it is particularly critical to find a ‘balance point’, that is, to promote collagen synthesis while minimising the impact on cell proliferation, to realize the optimization of the therapeutic effect of ‘promoting wound healing’ and ‘inhibiting scar production’. Meanwhile, given that all current studies are limited to in vitro cell experiments, additional human research is warranted.

In conclusion, CAP can solve the chronic stagnant state of DFUs by antibacterial and anti‐inflammatory actions, and promote the process of the wound to the proliferative and remodelling phases. Furthermore, it induces various signalling pathways to promote re‐epithelialisation, angiogenesis, matrix remodelling, and finally inhibits scar formation. The ‘dynamic and orderly’ natural healing is achieved.

## 4 Clinical Research of CAP in the Treatment of DFUs

Studies have shown that CAP plays a positive role in the healing of DFUs [116]. However, in actual clinical applications, DFUs are often accompanied by vascular neuropathy and other complications [117]. Therefore, conducting clinical trials is necessary. Although there are no authoritative clinical trial data available at present, an increasing number of clinical case studies indicate that CAP may have a beneficial effect on the healing of DFUs.

**FIGURE 8:**
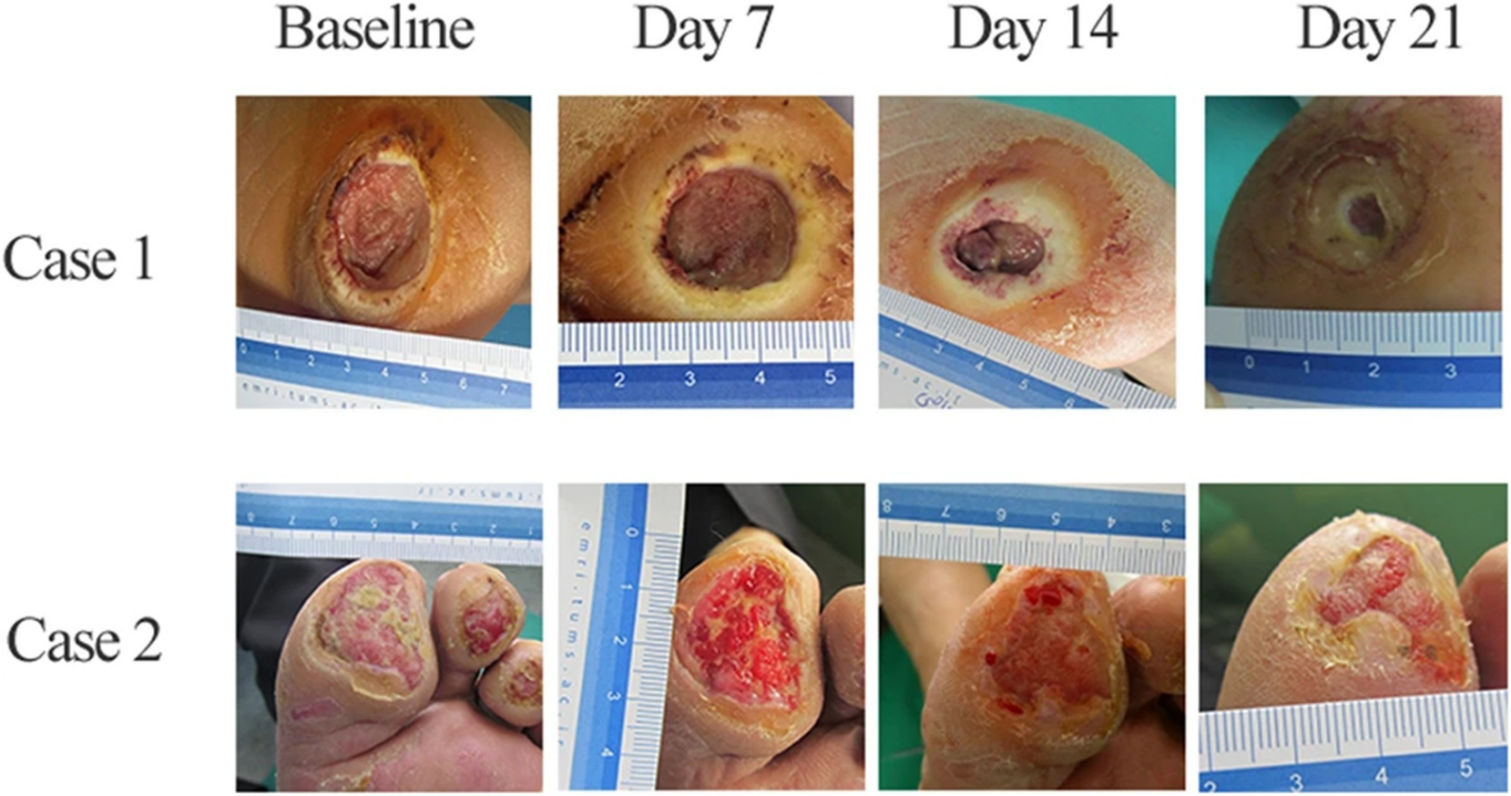
CAP can promote wound contraction for DFUs at two different sites. Reprinted from Ref. [28] with permission. Copyright 2020, The Author(s).

Stratmann et al., in a 2020 randomized clinical trial of DFUs comparing the use of kINPen Med with placebo as a control, showed that both treatments contracted the wound. At day 9, CAP treatment significantly increased wound healing (wound remaining area: CAP 30.5% vs. placebo 55.2%), and the total mean (SD) area of the wound was reduced. Moreover, it was proved that the effect of CAP on wound healing was not only dependent on the antibacterial effect but also promoted wound healing by activating the healing ability of DFUs [30]. Strohal et al. conducted a study in 2025, including 70 patients with chronic wounds (with diabetic patients accounting for 14.29%). The CAP group received 1–2 minutes of CAP treatment each time. The results showed that the average wound area of this group decreased by 0.70 cm^2^ per visit, while the control group decreased by an average of 0.36 cm^2^. This indicates that CAP treatment can significantly accelerate the healing speed of DFUs [118]. For clinical application, the acceleration of wound healing can greatly reduce the incidence of wound recurrence and the risk of amputation. Figure 8 demonstrates that CAP can promote healing of DFUs in different parts of the heel (Case 1) and forefoot (Case 2) [28].

CAP can also regulate the pH value of DFU, improve the microenvironment of the wound, and relieve the patient's pain. In a randomized, single‐blind, placebo‐controlled clinical trial by Strohal et al., it was observed that the pH value of 70 DFU patients at the last CAP treatment was significantly lower compared to that on the first day of treatment. This resulted in the wound recovering from a slightly alkaline environment to a normal acidic environment, which is conducive to wound healing [118]. In addition, CAP modifies the wound exudate to maintain an acidic environment for a longer period of time. At the same time, the pain is relieved. The patient was asked to rate the pain relief during the treatment using a VAS score. On the seventh day of treatment, the CAP treatment group had a score of 1, while the placebo treatment group still had a high score, proving that CAP was effective in pain relief [118].

CAP is anti‐inflammatory by reducing the levels of inflammatory cytokines, thereby promoting wound healing by playing the dual role of ‘antibacterial + anti‐inflammatory’. Amini et al. conducted a randomized controlled trial on DFUs, setting up a traditional treatment group (SC) and a traditional treatment combined with CAP treatment group (SC + CAP) for a comparative study. The results showed that the expression of inflammatory cytokines in both groups decreased after treatment, and the reduction in the SC + CAP group was significantly better than that in the SC group. This research result confirmed that CAP can effectively enhance the anti‐inflammatory effect of DFUs, providing experimental evidence for clinical inflammatory intervention. For bacterial load, the bacterial load of the SC + CAP group decreased significantly over time, which proved that CAP achieved wound healing by reducing inflammation and removing bacteria [32].

Up to now, as the clinical application of CAP in treating DFUs is in its infancy, there is still no CAP device specifically designed for the clinical treatment of DFUs; CAP has been fully applied in many fields, such as hospitals, dentistry, food, and agriculture, etc., and has demonstrated its outstanding antibacterial and other properties [119, 120, 121, 122] The technology is relatively mature, so the potential for transformation is huge.

## 5 Challenges and Prospects of CAP in the Treatment of DFUs

The above clinical trials have demonstrated the potential of CAP in promoting wound healing. However, there are still some challenges and deficiencies in the actual clinical application. Solving these problems is the key to promote the continuous development of CAP in the treatment of DFUs.

While CAP produces effective active substances, RONS, it also produces UV radiation, which is known to cause certain damage to the skin. Although many reports have proved that the UV radiation produced by a moderate amount of CAP is trace [123], it has been reported that the UV produced by long‐term CAP treatment plays a 70% role in DNA breakage and cell toxicity [124]. Therefore, when CAP is repeatedly used in clinical practice over a long period of time, ultraviolet radiation is likely to cause cytotoxicity. To eliminate the damage caused by ultraviolet radiation to cells and skin, two methods can be used in the future to address this issue. The first is to filter out most of the UV light by means of a device based on the linear propagation properties of light. But this approach may reduce the concentration of RONS entering the wound. The second is to develop a drug that absorbs UV light and apply it to the surface of the wound before performing CAP treatment. It should be noted that the drug can neither block the penetration of RONS to the site of action nor react with RONS [125].

**FIGURE 9:**
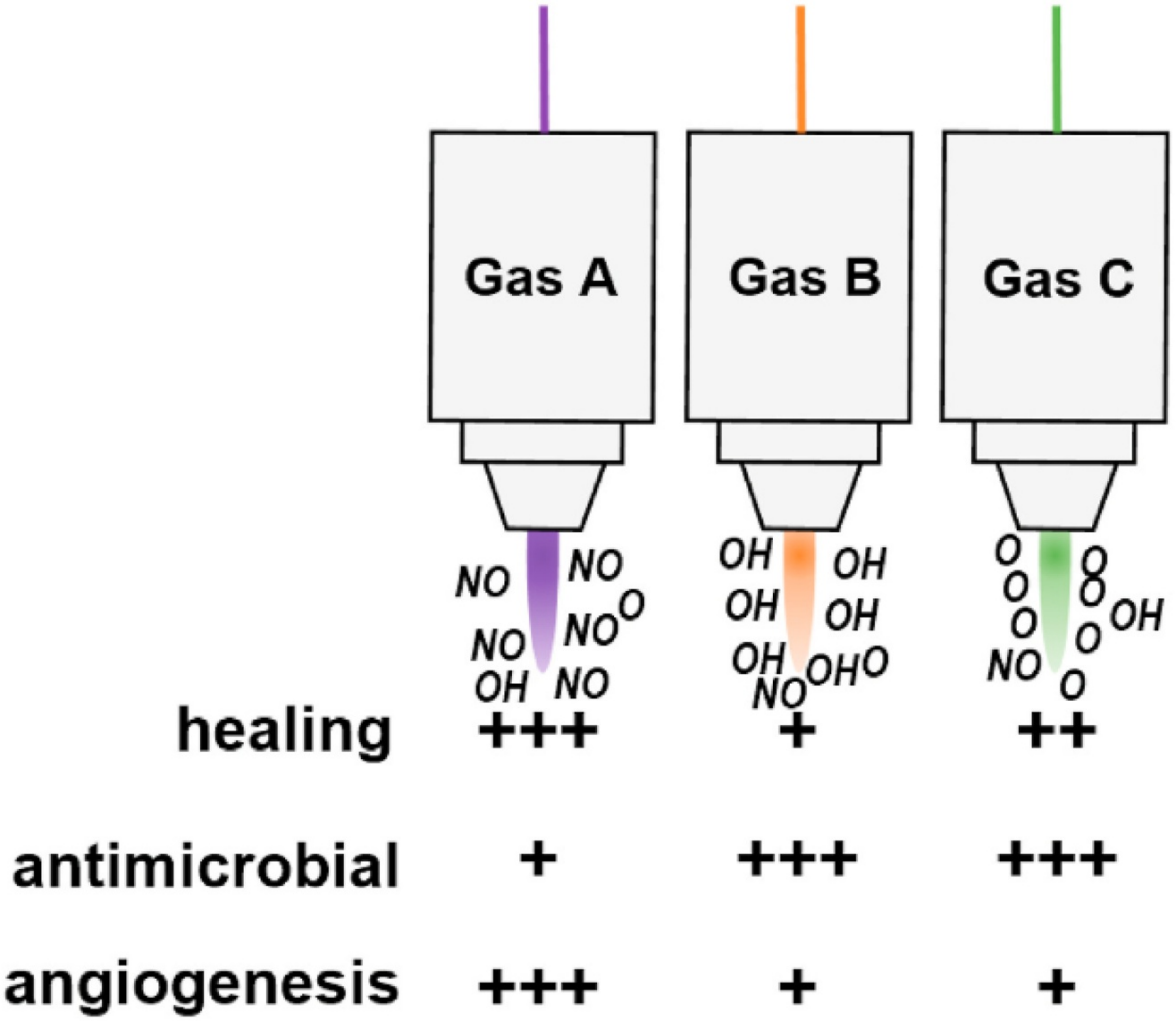
Schematic diagram of CAP devices driven by different working gases (Gas A, B, C) and the differences in their biological effects. The active species spectra produced by the three gases are different, resulting in different intensities of action in wound healing, antibacterial activity, and angiogenesis. Reprinted from Ref. [126] with permission. Published by Elsevier.

The lack of standardized CAP equipment and treatment parameters has significantly increased the uncertainty in its clinical application in DFUs. The CAP equipment models used in existing clinical studies vary, and there are significant differences in key parameters such as working gas, treatment duration, and treatment distance, which directly affect the antibacterial efficacy and wound healing effect (Figure 9). Due to the absence of unified equipment specifications and parameter standards at present, it is difficult to compare the clinical efficacy of each study horizontally and form a consistent conclusion, which is also an important reason why CAP, although its effect is clear, has insufficient clinical trial evidence at this stage. Therefore, in the future, by standardising and unifying the equipment data, linking the CAP equipment, exposure time and frequency, infection status, wound location, and the treatment site within the wound with objective responses, it will be beneficial for the development of CAP clinical trials and accelerate the transformation of CAP in the treatment of DFUs [126].

In addition to the issues of CAP equipment and the standardisation of treatment parameters, the high level of induced costs and insufficient accessibility are also worthy of attention. These two factors have limited the wide application of CAP in the treatment of DFUs. Expensive CAP equipment not only increases the operational costs of medical institutions, including ongoing maintenance of the equipment and training of professional personnel, but also raises the treatment expenses for patients, thereby reducing treatment compliance. Moreover, because of the high purchase cost of CAP instruments, grassroots clinics and small hospitals in remote areas usually cannot be equipped with the relevant equipment. For DFU patients, due to the inconvenience of moving the CAP equipment and the fact that they often have physical disabilities, this makes it difficult for patients in remote areas to complete the treatment course regularly, further reducing the clinical accessibility of this technology. Of course, the latest research has already developed portable devices powered by ambient air, but their efficacy in treating diabetic foot ulcers still needs further verification [127].

Although CAP has outstanding antibacterial properties, its effect on the balance of bacterial flora in the wounds cannot be ignored. The bacterial flora of DFUs is complex, and some probiotics also coexist with other bacteria. Although CAP has a broad spectrum of antibacterial properties and is effective in killing pathogenic bacteria, it may affect some probiotics at the same time, leading to the destruction of the balance of bacterial flora and further disorder of the wound microenvironment. At present, most studies focus on the killing effect of pathogenic bacteria, but there is a lack of research on the effect of CAP on probiotics, and whether CAP can selectively kill pathogenic bacteria is not known. Therefore, the balance of bacterial flora should be further studied.

In addition, the effect of CAP treatment alone is limited, and the combination of CAP with other therapies can expand more application scenarios. The pathology of DFUs is complex and affected by many factors. The single treatment of CAP alone cannot achieve high‐quality wound healing. Combined treatment can make up for this deficiency, which can better promote wound healing and also be suitable for more complex clinical scenarios.

**FIGURE 10:**
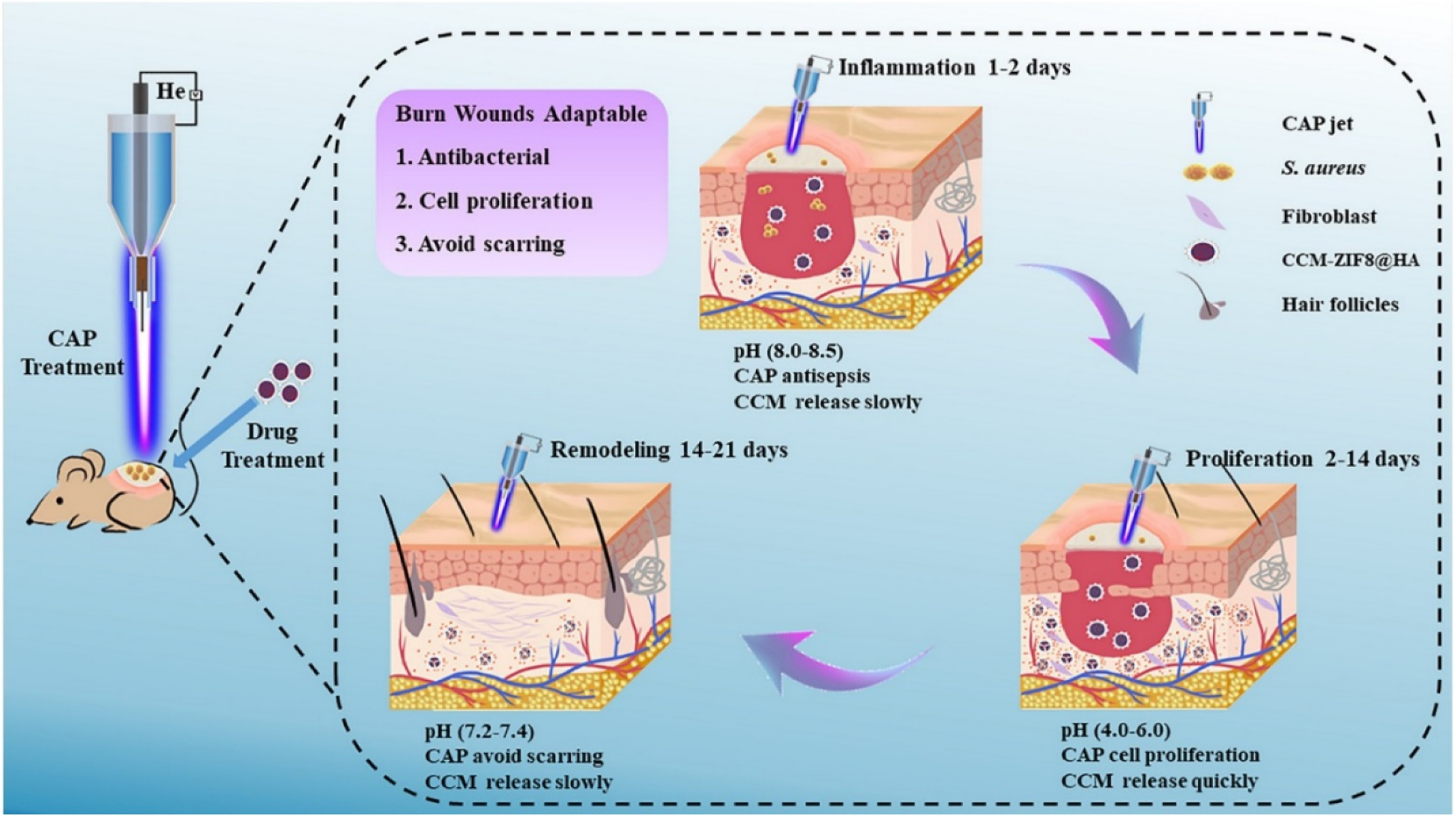
Schematic diagram of the adaptive release system combined with cold atmospheric plasma for burn treatment. Reprinted from Ref. [130] with permission. Copyright 2023, Science China Press.

For example, the high‐flow rate gas produced by CAP will dry the wound, and the drying of the wound is usually not conducive to wound healing [128, 129]. For how to perform CAP treatment while keeping the wound moist and not affecting the function of CAP, we put forward an idea: to combine hydrogel or other related materials with CAP. This can not only ensure wet wound healing but also play the role of hydrogel and CAP in wound healing [125]. However, it should be noted that due to the different crosslinking degrees of hydrogels, covering the wound surface is a ‘physical barrier’ to CAP, which affects the penetration rate of active substances. Therefore, this issue still needs further study in the future. For example, our team has developed a pH‐sensitive adaptive release system combined with CAP for the treatment of burn wounds [130] (Figure 10). This therapy achieves ‘on‐demand drug delivery’ by tailoring the actual drug needs for the three stages of wound healing. For the treatment of DFUs, because DFUs are affected by many pathological factors, they have a complex wound microenvironment. This combined therapy accurately corresponds to the complex microenvironment of DFUs and reduces the dose dependence of CAP. However, this treatment only focuses on promoting wound healing. Other complications of DFUs, such as hyperglycemia and vascular neuropathy, which can affect wound healing and recurrence, are not affected. If this therapy can improve complications while promoting wound healing, the clinical application scenario of CAP can be expanded, and it will have broader development prospects in the future.

## 6 Conclusions

In conclusion, CAP plays a comprehensive role in treating DFUs, covering nearly the entire wound healing process and showing unique advantages. Its excellent antibacterial effect not only effectively reduces the load of various bacterial types and breaks down biofilms but also prevents the development of drug resistance. The production of RONS can trigger the body's natural immune response, precisely regulate the expression of endogenous inflammation and growth factors, and facilitate the smooth progression of wound healing to the proliferation and remodelling phases. Additionally, it can promote wound re‐epithelialisation and angiogenesis without leaving scars. Significantly, all the above results were obtained through in vitro experiments. Although a small amount of clinical data is available to preliminarily support some of its effects, more authoritative clinical studies are needed for further validation. However, it also has certain drawbacks, including ultraviolet radiation emission, inconsistent equipment types and parameters, high induced costs, poor accessibility of CAP technology, induction of wound dryness, and potential disruption of the microbial community balance in the wound microenvironment. Of course, it is anticipated that through targeted approaches and further research, the inherent drawbacks of CAP can be addressed, thereby expediting its clinical translation. Through these approaches, it is hoped that CAP will see broader application in clinical practice. In summary, CAP, as a comprehensive and innovative treatment strategy, offers a new approach for managing DFUs.

## Author Contributions

**Yiran Wang:** writing the original draft. **Junxiao Zhang:** writing the original draft. **Jinting Yan:** writing – review and editing. **Wenhua Bi:** methodology, project administration. **Jinlong Ma:** writing – review and editing, methodology, project administration.

## Funding

This work was supported by the National Natural Science Foundation of China (Grant No. 82302840), the Shandong Provincial Natural Science Foundation (Grant No. ZR2023MH037), Shandong Provincial Traditional Chinese Medicine Science and Technology Project (Grant No. M‐2023084).

## Ethics Statement

The authors have nothing to report.

## Consent

All authors consent to publish.

## Conflicts of Interest

The authors declare no conflicts of interest.

## Data Availability

The data presented in this study are available in the referenced articles. The authors confirm that the data supporting the findings of this study are available within the paper and its online supplementary repository.
